# How Has Access to Care for Medi-Cal Enrollees Fared Relative to Employer-Sponsored Insurance 4 Years After the Affordable Care Act Expansion?

**DOI:** 10.1007/s11606-021-07383-3

**Published:** 2022-01-28

**Authors:** Susan H. Babey, Ninez A. Ponce, Tara Becker, Petra W. Rasmussen, A. J. Scheitler

**Affiliations:** 1 UCLA Center for Health Policy Research, University of California, Los Angeles, Los Angeles, CA USA; 2 Department of Health Policy and Management, Fielding School of Public Health, University of California, Los Angeles, Los Angeles, USA

**Keywords:** Medi-Cal, Medicaid, Employer-sponsored insurance, Access to care

## Abstract

**Background:**

The number of Californians covered by Medi-Cal increased more than 50% between 2013 and 2018, largely due to expansion under the Affordable Care Act (ACA). This rapid expansion of Medicaid rolls prompted concerns that Medi-Cal enrollees would face greater difficulty accessing health care.

**Objective:**

Examine whether gaps in access to care between Medi-Cal and employer-sponsored insurance (ESI) present in 2013 (prior to ACA implementation) had changed by 2018 (several years post implementation).

**Design:**

Secondary analysis of data from the 2013 and 2018 California Health Interview Survey. The sample included adults of ages 18–64 insured all year and covered by ESI or Medi-Cal at time of interview. Logistic regressions were used to examine variation across years in the association between access to care and insurance type.

**Main Measures:**

Five access to care outcomes were assessed: no usual source of care, not accepted as new patient in past year, insurance not accepted in past year, delayed medical care in past year, and difficulty getting timely appointment. The main predictors of interest were type of insurance (Medi-Cal or ESI) and survey year (2013 or 2018).

**Key Results:**

The association between insurance type and access to care changed significantly over time for three outcomes: not accepted as new patient in past year (OR = 0.55, 95% CI = 0.32–0.97), delayed medical care in past year (OR = 1.55, 95% CI = 1.06–2.25), and difficulty getting timely appointment (OR = 0.41, 95% CI = 0.23–0.74). Predicted probabilities indicate gaps between Medi-Cal and ESI narrowed for not accepted as new patient in past year and difficulty getting timely appointment, but widened for delayed medical care.

**Conclusions:**

Despite the rapid expansion in the number of Californians covered by Medi-Cal, most gaps in access to care between Medi-Cal and ESI enrollees improved or did not significantly change between 2013 and 2018.

## INTRODUCTION

Medi-Cal, California’s Medicaid program, serves as a critical component of the state’s health care safety net by providing health insurance coverage for Californians with low incomes. The number of Californians covered by Medi-Cal increased more than 50% from 8 million in 2013 to over 13 million in 2018, largely due to the program’s expansion under the Affordable Care Act (ACA).^1^ Nationally, total enrollment in Medicaid also increased significantly over this time period.^2^ In California, although enrollment in Medi-Cal increased sharply between 2013 and 2015, between 2016 and 2018, enrollment leveled off.^1,3,4^

The sharp increases between 2013 and 2015 in the number of people enrolled in Medi-Cal prompted concerns about the program’s ability to meet the health care needs of so many new enrollees. Access to care for Medicaid enrollees is better than that for the uninsured and improved for those who were uninsured and gained Medicaid coverage through ACA expansion.^5,6^ However, Medicaid enrollees have consistently reported lower access to care than those enrolled in employer-sponsored insurance (ESI).^7^

This study assesses changes over time in the quality of Medi-Cal participants’ access to health care by comparing access to care for Medi-Cal to ESI and determining whether the association between insurance type and access to care changed between 2013 (prior to implementation of the ACA) and 2018. The rapid expansion in Medi-Cal enrollment between 2013 and 2016 and subsequent stability in enrollment through 2018 make this time period particularly interesting to examine changes in any gaps in access to care between Medi-Cal and ESI.

## METHODS

### Data Source and Population

Data were from the adult sample of the California Health Interview Survey (CHIS) public use files from 2013 and 2017–2018. CHIS is a survey of households drawn from every county in California and is designed to be representative of California’s non-institutionalized population. A 2-stage, geographically stratified design with random-digit dialing of landlines and cell phones was used. One randomly selected adult (aged 18 or older) was interviewed in each household. Interviews were conducted in English, Spanish, Chinese, Vietnamese, Korean, and Tagalog (in 2017–2018). The adult response rate after the screening interview (in which survey is introduced and respondents are randomly selected) was 51.5% in 2013 and 42.3% in 2017–2018.^8,9^ Detailed descriptions of CHIS methodology are available elsewhere.^10,11^

A total of 20,724 adults completed the survey in 2013 and 42,330 in 2017–2018. To compare access to care for adults with Medi-Cal to those with ESI, our analytic sample was limited to adults of ages 18–64 who were insured for the entire year prior to being interviewed and who had Medi-Cal or ESI coverage at the time of interview. We excluded 23,946 adults of age 65+, 9377 with insurance other than ESI/Medi-Cal, and 1270 not continuously insured. This resulted in an analytic sample of 28,461.

### Measures

The primary predictors of interest were type of insurance at time of interview (Medi-Cal or ESI) and survey year (2013 or 2018). Five indicators of access to care were examined as outcomes: (1) no usual source of care other than emergency room, (2) not accepted as new patient by a doctor in past year, (3) insurance not accepted by doctor in past year, (4) delaying or foregoing needed medical care in past year, and (5) not able to get a timely appointment in past year. The last indicator was limited to 9419 adults who reported trying to get an appointment within the next 2 days due to sickness or injury. These respondents were asked about scheduling the appointment. We selected these measures to inform health care system enabling opportunities for the Medi-Cal program to improve access for beneficiaries. The first three measures capture difficulties in making connections to health care systems. The last two measures demonstrate gaps in receiving needed health care.

Our analytic approach follows Long et al.’s use of the Andersen Behavioral Model by augmenting population adjustments based on an individual’s health care need, which is conditioned by age, gender, health status, and disability status, with social risk factors that shape access to health care: race/ethnicity, education, English proficiency, income, and rural/urban status.^6,12^ Adjustments for these factors increase the comparability of individuals enrolled in Medi-Cal with those in ESI in empirical models.

The following characteristics were included as covariates: age (continuous), gender, race/ethnicity (Latino, non-Latino White, non-Latino Asian, non-Latino Black or African-American, non-Latino American Indian, and a combined category of non-Latino Native Hawaiian/Pacific Islander, non-Latino other race and non-Latino two or more races), income, education level (less than high school, high school graduate, college graduate or higher), English proficiency (limited English proficiency, speaks English well, or speaks English very well combined with speaks only English), and living in an urban or rural area. Household income was examined as percent of the federal poverty level (below 100%, 100–199%, 200–299%, 300% and above). This variable is a ratio of household income to federal poverty threshold (which varies by household size) and is constructed based on household size, household income, and U.S. Census Bureau poverty thresholds. Two health indicators were included: self-reported health status was categorized into excellent/very good, good, and fair/poor, and self-reported receipt of social security disability income (yes or no) was included as a proxy for long-term disability.

### Analyses

Logistic regression analyses were used to examine the association of insurance type with access to care adjusting for the covariates discussed above. Models included year-by-insurance type interactions to test for variations over time in the association between insurance type and access indicators. Post-estimation predicted probabilities were estimated to determine the magnitude of the changes. A difference-in-differences(DID) estimate measuring the net percentage point change between Medi-Cal and ESI over time was estimated, and we applied the delta method to test the significance of the DID estimate. A significant positive DID indicates widening gaps in access over time, whereas a significant negative DID indicates narrowing gaps. CHIS data from 2017 and 2018 were pooled, and weights representing the 2018 California population were applied. Thus, we refer to this year as 2018 throughout the paper. Survey weights are applied to adjust for non-response and survey design effects and to ensure weighted estimates are representative. Several dimensions are used in survey weight development: demographics (age, sex, race, and ethnicity), geographic variables (county), household composition (presence of children in the household), and socio-economic variables (home ownership and education). The weighted sample was shown to be representative of California’s population not living in correctional or congregate housing facilities.^13^ Analyses were conducted using SAS 9.4 and Stata 16.0.

## RESULTS

Table 1 displays characteristics of the study population and outcome measures stratified by year and insurance type. Changes in the study population composition were the result of both demographic shifts in California and changes in the populations enrolled in Medi-Cal and ESI. The proportion Latino increased between 2013 and 2018, with a corresponding decrease in the proportion non-Latino White among both Medi-Cal and ESI enrollees. Due to Medi-Cal expansion to all low-income adults (excluding those who are undocumented), Medi-Cal enrollees comprised a larger proportion of the sample in 2018 than in 2013 (32.7% vs 18.2%). A smaller proportion of Medi-Cal enrollees had incomes below 100% federal poverty level (FPL) in 2018 than in 2013, whereas this proportion was slightly larger in 2018 among ESI enrollees. The proportion in fair or poor health decreased among Medi-Cal enrollees but increased among ESI enrollees.

**Table 1:** Population Characteristics of California Adults, Ages 18–64, Insured All Year and Insured by Medi-Cal or ESI at Time of Interview

	2013				2018			
	(*N* = 8776)				(*N* = 19,685)			
	Medi-Cal		ESI		Medi-Cal		ESI	
	%	SE	%	SE	%	SE	%	SE
Age								
18–34	40.23	2.01	32.1	0.78	45.13	1.72	31.61	0.68
35–49	32.35	2.2	35.02	0.79	29.3	0.94	34.84	0.94
50–64	27.42	1.59	32.88	0.62	25.56	1.37	33.55	0.58
Gender								
Male	43.97	2.27	48.96	0.77	43.82	1.29	50.6	0.67
Female	56.03	2.27	51.04	0.77	56.18	1.29	49.4	0.67
Race/ethnicity								
Latino	55.58	2.25	28.32	0.77	57.96	2.52	29.18	1.37
Black or African-American	10.7	1.45	4.93	0.33	6.75	0.59	5.08	0.99
American Indian or Alaska Native	0.76	0.25	0.45	0.09	0.64	0.16	0.25	0.07
Asian	6.39	1.23	17.86	0.71	10.82	2.57	17.46	2.61
Other	2.75	0.67	2.35	0.2	2.69	0.64	2.8	0.42
White	23.82	1.84	46.09	0.84	21.13	1.18	45.22	1.03
Income (as percent of federal poverty level)								
0–99% FPL	53.96	2.3	3.5	0.45	39.92	2.25	4.22	0.83
100–199% FPL	29.57	2.16	11.9	0.86	33.43	1.49	8.07	0.49
200–299% FPL	9.41	1.26	12.7	0.57	13.48	0.97	11.61	1.6
300% FPL and above	7.06	1.15	71.9	0.82	13.18	1.64	76.1	1.13
Educational attainment								
Less than high school	33.67	1.8	7.27	0.49	30.9	1.06	6.32	0.85
High school graduate	58.44	1.98	44.71	1.07	53.57	3.37	38.63	1.44
College graduate or higher	7.89	1.22	48.02	1.07	15.53	2.73	55.05	0.93
English proficiency								
English only/very well	56.2	2.55	82.46	0.78	61.69	2.88	85.68	0.66
Well	16.5	1.93	10.38	0.68	12.94	0.82	8.47	0.69
Limited English proficiency	27.3	2.05	7.16	0.64	25.36	3.15	5.85	0.81
Lives in urban or rural area								
Urban	88.82	1.11	91.53	0.52	88.46	2.16	91.4	0.38
Rural	11.18	1.11	8.47	0.52	11.54	2.16	8.6	0.38
Health status								
Excellent or very good	28.41	2.15	61.39	1.02	31.41	2.17	58.69	1.38
Good	34.64	2.25	27.84	0.97	35.07	2.91	29.97	1.26
Fair or poor	36.95	2.07	10.77	0.74	33.52	1.48	11.35	0.55
Receiving SSDI								
Yes	20.84	1.57	1.21	0.21	11.42	0.87	0.84	0.14
No	79.16	1.57	98.79	0.21	88.58	0.87	99.16	0.14
Has usual source of care								
Yes	82.48	2.03	92.16	0.7	79.05	1.49	91.77	0.65
No	17.52	2.03	7.84	0.7	20.95	1.49	8.23	0.65
Not accepted as new patient by doctor, past year								
Yes	6.41	1.28	2.17	0.21	5.11	0.58	3.3	0.34
No	93.59	1.28	97.83	0.21	94.89	0.58	96.7	0.34
Insurance not accepted by doctor, past year								
Yes	8.41	1.38	2.36	0.29	8.64	0.61	3.4	0.92
No	91.59	1.38	97.64	0.29	91.36	0.61	96.6	0.92
Delayed medical care, past year								
Yes	14.98	1.9	14.44	0.72	16.44	1.17	11.95	0.47
No	85.02	1.9	85.56	0.72	83.56	1.17	88.05	0.47
Able to get timely appointment, past year (among those who sought)								
Yes	82.81	2.93	94.49	0.78	82.61	1.9	86.65	1.65
No	17.19	2.93	5.51	0.78	17.39	1.9	13.35	1.65

Source: 2013 and 2017–2018 pooled California Health Interview Survey. CHIS data from 2017 and 2018 were pooled, and weights representing the California population in 2018 were applied. Thus, we refer to this year as 2018 throughout the paper

**Figure 1:**
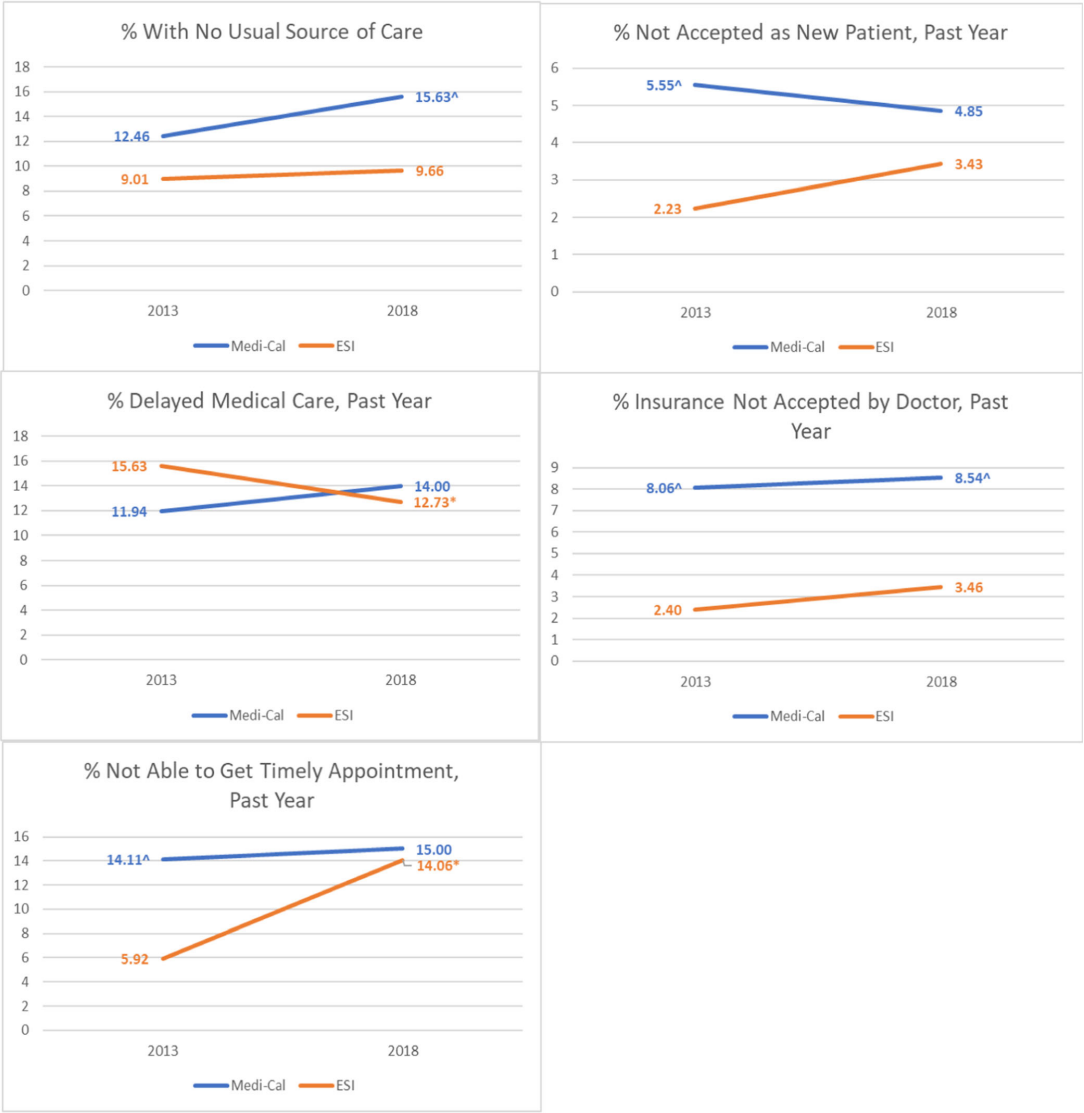
Adjusted predicted probabilities for each access indicator, California adults of ages 18–64, insured all year and insured by Medi-Cal or ESI at time of interview. Blue line, Medi-Cal; orange line, ESI; asterisk, significantly different from 2013; caret, significantly different from ESI. ESI = employer-sponsored insurance. CHIS data from 2017 and 2018 were pooled, and weights representing the California population in 2018 were applied. Thus, we refer to this year as 2018 throughout the paper. Source: 2013 and 2017–2018 pooled California Health Interview Survey

Table 2 and Figure 1 show predicted probabilities for each access indicator as a function of insurance type and year adjusting for sociodemographic and health covariates. Between 2013 and 2018, the percent with no usual source of care increased from 12.5 to 15.6% for Medi-Cal and from 9.0 to 9.7% for ESI (aOR: 1.23, 95% CI: 0.77–1.94). The percent not accepted as new patient decreased from 5.6 to 4.9% for Medi-Cal and increased from 2.2 to 3.4% for ESI (aOR: 0.55, 95% CI: 0.32–0.97). The percent whose insurance was not accepted increased from 8.1 to 8.5% for Medi-Cal and increased from 2.4 to 3.5% for ESI (aOR: 0.73, 95% CI: 0.34–1.56). The percent who reported delaying medical care increased from 11.9 to 14.0% for Medi-Cal and decreased from 15.6 to 12.7% for ESI (aOR: 1.55, 95% CI: 1.06–2.25). The percent not able to get a timely appointment went from 14.1 to 15.0% for Medi-Cal and increased from 5.9 to 14.1% for ESI (aOR: 0.41, 95% CI: 0.23–0.74).

**Table 2:** Adjusted Probability for Each Access Indicator as a Function of Year and Insurance Type, California Adults Ages 18–64, Insured All Year and Insured by Medi-Cal or ESI at Time of Interview

	**2013**		**2018**		**2018–2013**
	%	95% CI	%	95% CI	
**No usual source of care**					
Medi-Cal	12.46	8.93–16.00	15.63	11.38–19.89	3.17
ESI	9.01	7.37–10.65	9.66	8.28–11.03	0.65
Medi-Cal-ESI	3.45		5.97		**2.52**
**Not accepted as new patient by doctor, past year**					
Medi-Cal	5.55	2.71–8.39	4.85	3.45–6.25	− 0.70
ESI	2.23	1.78–2.68	3.43	2.54–4.32	1.20
Medi-Cal-ESI	3.32		1.42		− 1.90*
**Insurance not accepted by doctor, past year**					
Medi-Cal	8.06	4.62–11.51	8.54	6.15–10.94	0.48
ESI	2.40	1.81–2.98	3.46	1.35–5.56	1.06
Medi-Cal-ESI	5.66		5.98		**− 0.58**
**Delayed medical care, past year**					
Medi-Cal	11.94	8.62–15.27	14.00	12.06–15.95	2.06
ESI	15.63	14.07–17.2	12.73	11.66–13.80	− 2.9
Medi-Cal-ESI	− 3.69		1.27		4.96*
**Not able to get timely appointment, past year**					
Medi-Cal	14.11	8.25–19.97	15.00	10.11–19.89	0.89
ESI	5.92	4.17–7.67	14.06	9.98–18.14	8.14
Medi-Cal-ESI	8.19		0.94		− 7.25*

Source: 2013 and 2017–2018 pooled California Health Interview Survey. CHIS data from 2017 and 2018 were pooled, and weights representing the California population in 2018 were applied. Thus, we refer to this year as 2018 throughout the paper. Bolded estimates are difference in differences

*ESI*employer-sponsored insurance

*Significant at *p* < .05

Table 3 displays the logistic regression results adjusted for covariates shown. Year-by-insurance type interactions were significant for three access indicators suggesting gaps in access to care between Medi-Cal and ESI changed between 2013 and 2018 for the following indicators: not accepted as new patient in past year, unable to get timely appointment in the past year, and delayed or did not get needed medical care in past year. Additionally, women were more likely to not be accepted as a new patient or to delay care and those with fair or poor health status were more likely to delay care and to have difficulty getting a timely appointment.

**Table 3:** Logistic Regressions Testing Year-by-Insurance Type Interaction in Models of Access to Care Indicators, California Adults Ages 18–64, Insured All Year and Insured by Medi-Cal or ESI at Time of Interview

	**No usual source of care**		**Not accepted as new patient by doctor, past year**		**Insurance not accepted by doctor, past year**		**Delayed medical care, past year**		Not able to get timely appointment, past year^*^	
	AOR	95% CI	AOR	95% CI	AOR	95% CI	AOR	95% CI	AOR	95% CI
**Year-by-insurance type interaction**	1.23	(0.77–1.94)	**0.55**	**(0.32**–**0.97)**	0.73	(0.34–1.56)	**1.55**	**(1.06**–**2.25)**	**0.41**	**(0.23**–**0.74)**
Year (ref = 2018)										
2013	0.75	(0.52–1.10)	1.15	(0.71–1.88)	0.94	(0.62–1.42)	0.83	(0.58–1.17)	0.93	(0.58–1.49)
Insurance type (ref = Medicaid)										
ESI	**0.55**	**(0.39**–**0.78)**	0.69	(0.43–1.13)	**0.38**	**(0.16**–**0.93)**	0.89	(0.72–1.1)	0.93	(0.47–1.81)
**Covariates**										
Age (continuous)	**0.96**	**(0.95**–**0.97)**	1.00	(0.99–1.01)	1.00	(0.99–1.01)	1.00	(0.99–1.01)	0.99	(0.98–1.00)
Gender (ref = male)										
Female	**0.47**	**(0.38**–**0.57)**	**1.49**	**(1.15**–**1.93)**	1.25	(0.94–1.66)	**1.49**	**(1.18**–**1.89)**	1.13	(0.66–1.93)
Race/ethnicity (ref = white)										
Latino	1.05	(0.85–1.28)	0.62	(0.36–1.08)	0.73	(0.36–1.48)	**0.79**	**(0.67**–**0.94)**	1.12	(0.67–1.84)
Black	1.17	(0.49–2.78)	0.76	(0.34–1.72)	0.75	(0.42–1.34)	**0.72**	**(0.52**–**0.99)**	1.12	(0.57–2.18)
AIAN	0.82	(0.25–2.71)	0.42	(0.09–1.99)	0.90	(0.19–4.32)	1.46	(0.59–3.62)	1.59	(0.12–21.09)
Asian	1.05	(0.78–1.41)	1	(0.63–1.59)	0.85	(0.51–1.41)	**0.67**	**(0.51**–**0.89)**	0.81	(0.49–1.34)
Other	1.1	(0.41–2.93)	1.53	(0.83–2.85)	1.44	(0.83–2.49)	0.99	(0.59–1.68)	1.49	(0.67–3.34)
Income (ref = 300% FPL and above)										
0–99% FPL	1.38	(0.81–2.34)	1.14	(0.77––1.71)	1.30	(0.86–1.96)	**1.60**	**(1.23**–**2.07)**	1.28	(0.52–3.16)
100–199% FPL	1.35	(0.95–1.92)	1.06	(0.67–1.67)	1.13	(0.54–2.36)	1.07	(0.78–1.46)	1.10	(0.76–1.58)
200–299% FPL	1.18	(0.71–1.97)	0.94	(0.51–1.71)	1.55	(1.00–2.42)	1.09	(0.83–1.45)	1.36	(0.89–2.08)
Educational attainment (ref = college graduate or higher)										
Less than high school	**1.8**	**(1.32**–**2.44)**	0.88	(0.46–1.69)	0.86	(0.41–1.80)	1.09	(0.69–1.71)	1.06	(0.60–1.90)
High school graduate	0.99	(0.66–1.47)	1.49	(0.76–2.90)	1.18	(0.75–1.85)	1.02	(0.71–1.46)	1.01	(0.61–1.67)
English proficiency (ref = English only/very well)										
Limited English proficiency	**1.94**	**(1.06**–**3.55)**	0.87	(0.13–5.68)	0.71	(0.08–6.66)	**0.38**	**(0.24**–**0.61)**	0.47	(0.17–1.31)
Speaks English well	0.99	(0.66–1.49)	1.14	(0.70–1.86)	0.81	(0.49–1.35)	**0.60**	**(0.45**–**0.81)**	0.79	(0.36–1.71)
Lives in urban or rural area (ref = urban)										
Rural	1.03	(0.67–1.59)	1.41	(0.62–3.23)	1.09	(0.77–1.54)	0.98	(0.71–1.36)	1.06	(0.75–1.52)
Health status (ref = excellent/very good)										
Fair or poor	0.98	(0.75–1.27)	1.28	(0.83–1.97)	1.46	(0.90–2.37)	**2.44**	**(1.98**–**3.01)**	**1.77**	**(1.18**–**2.66)**
Good	0.98	(0.82–1.18)	0.99	(0.66–1.50)	1.07	(0.82–1.40)	**1.59**	**(1.32**–**1.92)**	**1.44**	**(1.03**–**2.02)**
Receiving SSDI (ref = no)										
Yes	0.83	(0.55–1.26)	**1.99**	**(1.13**–**3.52)**	1.04	(0.60–1.78)	1.05	(0.76–1.45)	0.93	(0.51–1.72)

Source: 2013 and 2017–2018 pooled California Health Interview Survey. CHIS data from 2017 and 2018 were pooled, and weights representing the California population in 2018 were applied. Thus, we refer to this year as 2018 throughout the paper. Bold type indicates significant association, *p* < 0.05

*AIAN* American Indian or Alaska Native

*Limited to adults who sought an appointment within 2 days

On most measures, Medi-Cal enrollees reported lower access to care than ESI enrollees in 2013. In 2013, Medi-Cal enrollees were significantly more likely than those with ESI to have no usual source of care other than the emergency room, to not be accepted as a new patient, and to not have their insurance accepted. In addition, Medi-Cal enrollees were twice as likely to have had difficulty getting a timely appointment in the past year. Despite these gaps in access, Medi-Cal enrollees were less likely to have delayed receiving needed medical care than those with ESI.

In 2018, Medi-Cal enrollees remained significantly more likely than ESI enrollees to have no usual source of care and to not have their insurance accepted. However, there was no longer a statistically significant difference between Medi-Cal and ESI in the percent not accepted as new patients or that had difficulty getting a timely appointment. Though Medi-Cal enrollees were less likely than ESI enrollees to have delayed care in the past year in 2013, by 2018, they were more likely than those with ESI to do so.

The final column in Table 3 presents the difference-in-differences estimates showing the changes over time in gaps in access between Medi-Cal and ESI with positive values indicating widening gaps between Medi-Cal and ESI. Gaps in access between Medi-Cal and ESI changed for three of five outcomes in this study. Medi-Cal significantly improved relative to ESI on two measures—not accepted as new patient (− 1.90 percentage points) and not being able to get a timely appointment (− 7.25 percentage points)—but experienced a growing gap on delaying needed medical care (4.96 percentage points).

## DISCUSSION

Medi-Cal serves as a critical health safety net for more than 13 million Californians. Although research suggests access to care for those with Medi-Cal is better than for the uninsured, gaps exist between those with Medi-Cal and those with private insurance, particularly ESI.^6^ We examined whether these gaps in access to care changed between 2013, prior to implementation of the ACA, and 2018, several years after implementation.

Our findings suggest that access to care within Medi-Cal improved relative to ESI between 2013 and 2018 on some access indicators but not others. When this occurred, it was due less to improvements over time in access to Medi-Cal than to declining access to care among those with ESI. Notably, the narrowed gap between Medi-Cal and ESI on difficulty getting a timely appointment is due almost entirely to an increase among ESI enrollees (from 6 to 14%). More research is needed to understand this increase for ESI as there was little change for Medi-Cal enrollees. Despite the large increase in Medi-Cal enrollment after the ACA’s coverage expansion, the proportion of Medi-Cal enrollees that were told a doctor was not accepting new patients or who were not able to get a timely appointment did not change significantly after adjusting for changes in the Medi-Cal population. Instead, declines in access among ESI enrollees played a larger role in declining gaps between Medi-Cal and ESI. This suggests that changes in access to care are not specific to Medi-Cal but associated with a broader shift in accessibility of health care within California. However, the fact that Medi-Cal coverage expanded so dramatically within a short time period without leading to a corresponding erosion in access to care should not be ignored.

One exception occurred among the percentage who delayed needed medical care in the past year. The percentage who delayed care among Medi-Cal enrollees increased slightly, while decreasing among those with ESI, leading to a significant increase in the gap between Medi-Cal and ESI. It is notable that this occurred despite the lack of change in Medi-Cal enrollees’ ability to find a doctor that accepts new patients and/or accepts their health insurance and to make an appointment with their doctor in a timely manner. This suggests that these delays in care derive from a source other than the failure of connections with the health care system. It is possible that the expansion of Medi-Cal to the long-term uninsured might have led to different health care use patterns that may dissipate over time as these populations learn to navigate the health care system.

Though the change was not significant, the results also show an increase in the proportion of Medi-Cal enrollees who report having no usual source of care. This could be due to new enrollment of the previously long-term uninsured population and might indicate difficulties these populations face in creating connections to the health care system.^14,15^ Other research suggests that the newly insured experience barriers to care including problems navigating the health system, not knowing how to use coverage, cost concerns, or difficulty finding a provider.^15,16^ Enabling connections to the health care system is important for long-term health outcomes of these populations. Those with a usual source of care are more likely to seek preventive treatment, which can lead to fewer hospitalizations and medical costs in the future.^17^

In most cases, improved access for Medi-Cal enrollees relative to ESI was driven by declines in access among ESI enrollees. While enrollment in Medi-Cal was considerably higher in 2018 than 2013, the proportion with ESI did not differ between 2013 and 2018.^18^ It is unlikely that worse access among ESI enrollees was due to decreases in the proportion with employer coverage. Rather, other factors likely influenced health care access within California. For example, health literacy has been associated with delaying health care and difficulty finding a provider, and adults with public insurance, like Medi-Cal, are more likely to have lower health literacy.^19,20^ It is also possible that new Medi-Cal enrollees delay care due to cost concerns because they may not realize there are no copays, deductibles, or out-of-pocket payments.

It is worth noting that after 2018, enrollment in Medi-Cal began declining but then increased again in 2020–2021, likely due to economic impacts of the pandemic and rules preventing eligibility redeterminations.^4,21^ Although the present study used data collected prior to the Covid-19 pandemic, the overall finding that sharp increases in Medi-Cal enrollment were not associated with worse access to care for Medi-Cal enrollees suggests that Medi-Cal is an asset that may have helped mitigate some of the economic impacts of the pandemic in California.

This study has some limitations. First, while individuals had to be insured continuously for the past year to be included, they did not have to be insured with the same coverage type. This means that individuals could have been enrolled in a different source of coverage at the time of any gaps that they reported. However, restricting the sample to respondents with continuous health insurance coverage reduces the likelihood of churn in this sample. Medi-Cal coverage is renewed annually, although renewal is automatic for most. Second, our analysis was based on data from a single state and the findings may not extend to the experience of enrollees in other states. Third, the outcomes we examined rely on self-report and may be subject to recall bias or error. Finally, California prepared for the implementation of the ACA’s Medicaid expansion, through its Low Income Health Program (LIHP). Starting in 2010, LIHP allowed counties to expand coverage to adults with incomes below 138% FPL before the federal Medicaid expansion went into effect. Nearly 500,000 Californians participated in LIHP. This early expansion of Medi-Cal helped boost enrollment in Medicaid and prepare Californians for coverage protections offered by the ACA.^22^ As a result, a comparison between 2013 and 2018 may not fully capture pre- and post-expansion. However, there was still a 50% increase in Medi-Cal enrollment between 2013 and 2018 (5 million more enrollees), so the current analysis still provides a useful assessment of how gaps in access to care may have changed following a large influx of enrollees.

Despite the rapid expansion in the number of Californians covered by Medi-Cal, most gaps in access to care between Medi-Cal and ESI enrollees improved or did not significantly change between 2013 and 2018. However, when gaps between Medi-Cal and ESI improved, this tended to occur because of declines in access to care among those with ESI, and gaps on delays in care widened. Thus, our findings broadly suggest that there is room for improving connections to the health system—ensuring a usual source of care, increasing the supply of providers that will take Medi-Cal patients, and incentivizing providers to see Medi-Cal patients. Some of these connections to the health care system were more favorable for Medi-Cal enrollees in 2018 than they were in 2013. Strengthening health care system connections could reverse the troubling trend of widening disparities between Medi-Cal and ESI in delays or foregone needed medical care. Policy improvements in these access-to-care areas are critical for timely and appropriate care, and would improve the health and well-being of the 13 million Californians covered by Medi-Cal. Nevertheless, the fact that Medi-Cal coverage expanded so dramatically within a short time period without leading to a corresponding erosion in access to care suggests that access to care for Medi-Cal enrollees was not significantly negatively impacted by the sharp increase in enrollment.
